# Systemically Achievable Doses of Beer Flavonoids Induce Estrogenicity in Human Endometrial Cells and Cause Synergistic Effects With Selected Pesticides

**DOI:** 10.3389/fnut.2021.691872

**Published:** 2021-06-07

**Authors:** Georg Aichinger, Gloria Bliem, Doris Marko

**Affiliations:** ^1^Department for Food Chemistry and Toxicology, Faculty of Chemistry, University of Vienna, Vienna, Austria; ^2^Laboratory of Toxicology, Department of Health Sciences and Technology, Eidgenoessische Technische Hochschule (ETH) Zurich, Zurich, Switzerland

**Keywords:** endocrine disruptors, phytoestrogen, mixture effect, antioxidant, synergism, xenoestrogen, functional food, beer

## Abstract

Some prenylated polyphenols originating from hops, which are thus natural constituents of beer, have been discussed critically for their agonistic potential toward estrogen receptors. So far, little attention has been attributed to the fact that humans are typically not exposed to isolated compounds, but to mixtures which for example might comprise in addition to hop flavonoids further xenoestrogens, e.g., certain pesticides used for plant protection of hops and barley. Thus, we used the alkaline phosphatase assay to assess combinatory estrogenic effects of three signature compounds – xanthohumol, 8-prenylnaringenin and iso-xanthohumol–on Ishikawa cells in a combination that resembled the concentration ratios observable in beer. Moreover, we added this natural flavonoid pattern to a mixture of representative estrogenic pesticides to assess their combined effects. Using state-of-the-art statistical tools, we observed cumulative to slightly synergistic effects between isolated flavonoids as well as the flavonoid and the pesticide mixture. Of potential importance, these effects were found at low nanomolar hop polyphenol concentrations that one can reasonably expect to occur *in vivo* after the consumption of strongly hopped beer. Taken together, our results imply that cumulative/synergistic estrogenicity should be explored in detail and urgently be incorporated into risk assessment of prenylated chalcones.

## Introduction

Endocrine disruptive chemicals (EDCs) are in the spotlight of an ongoing public debate. Particularly, the impact of exposure to so-called “xenoestrogens,” exogenous compounds that target the human estrogen receptor (ER), on human health is extensively discussed (1, 2). The public debate has contributed to the advance of regulative limits for food-contaminating chemicals such as bisphenol A, phthalates, and several pesticides. Among the latter, the 1970's scandal around the insecticide and endocrine disruptor dichlorodiphenyltrichlorethane (DDT) has cumulated in its world-wide ban with the Stockholm convention of 2001 (3). While the European Union has since forbidden the new registration of hormonally active compounds for agricultural use (4), such chemicals are still applied on fields in other parts of the world, including the USA and Canada. Without a doubt, the regulations issued within the EU have improved consumer safety. However, recent research makes way for the suspicion that risk assessment for xenoestrogens is still incomplete, as it (a) yet lacks the incorporation of mixture effects and (b) might thus seriously underestimate the impact of xenoestrogens occurring in food as natural constituents.

“Mixture effects” or “combinatory effects” are defined as the biological impact caused by two or more chemicals, and can be divided in two groups: A “cumulative” or “additive” effect describes the event when several chemicals cause a combinatory effect that can be explained by addition of their effects as single compounds (5). If this is not the case, a mixture effect is referred to as “interaction,” which can in turn be either antagonistic (the combinatory effect is lower than the additive effect of the single compounds) or synergistic (vice versa).

Concerning pesticides, the European Food Safety Authority (EFSA) is working on a cumulative risk assessment program (6), from which two pilot studies are published, focusing on pesticides affecting the thyroid (7) and the nervous system (8). However, important this initiative is for consumer safety, it primarily focuses on cumulative effects rather than synergisms, and moreover, does not consider combinatory effects with potentially co-occurring bioactives from other sources than plant protection agents. Particularly in the case of estrogenicity, this might lead to an underestimation of the chemical hazard.

Fueled by the comparably unspecific ligand binding of ERs, the sheer amount of known xenoestrogens from a wide range of different - including natural - sources makes their co-occurrence in food the rule rather than the exception (2). Therefore, it seems reasonable to expect combinatory effects on the endocrine system. This is also reflected in recent literature. Vejdovszky et al. (9) described synergistic estrogenic effects of co-occurring mycotoxins as well as a synergistic estrogenic interaction of the soy flavonoid genistein with single representatives of those natural food contaminants (10). Furthermore, we recently described cumulative estrogenic effects of mycotoxins with the controversially discussed endocrine disruptor bisphenol A (11) and an antagonistic interaction between regulated *Fusarium* toxins and a mixture of hop polyphenols (12).

**Figure 1:**
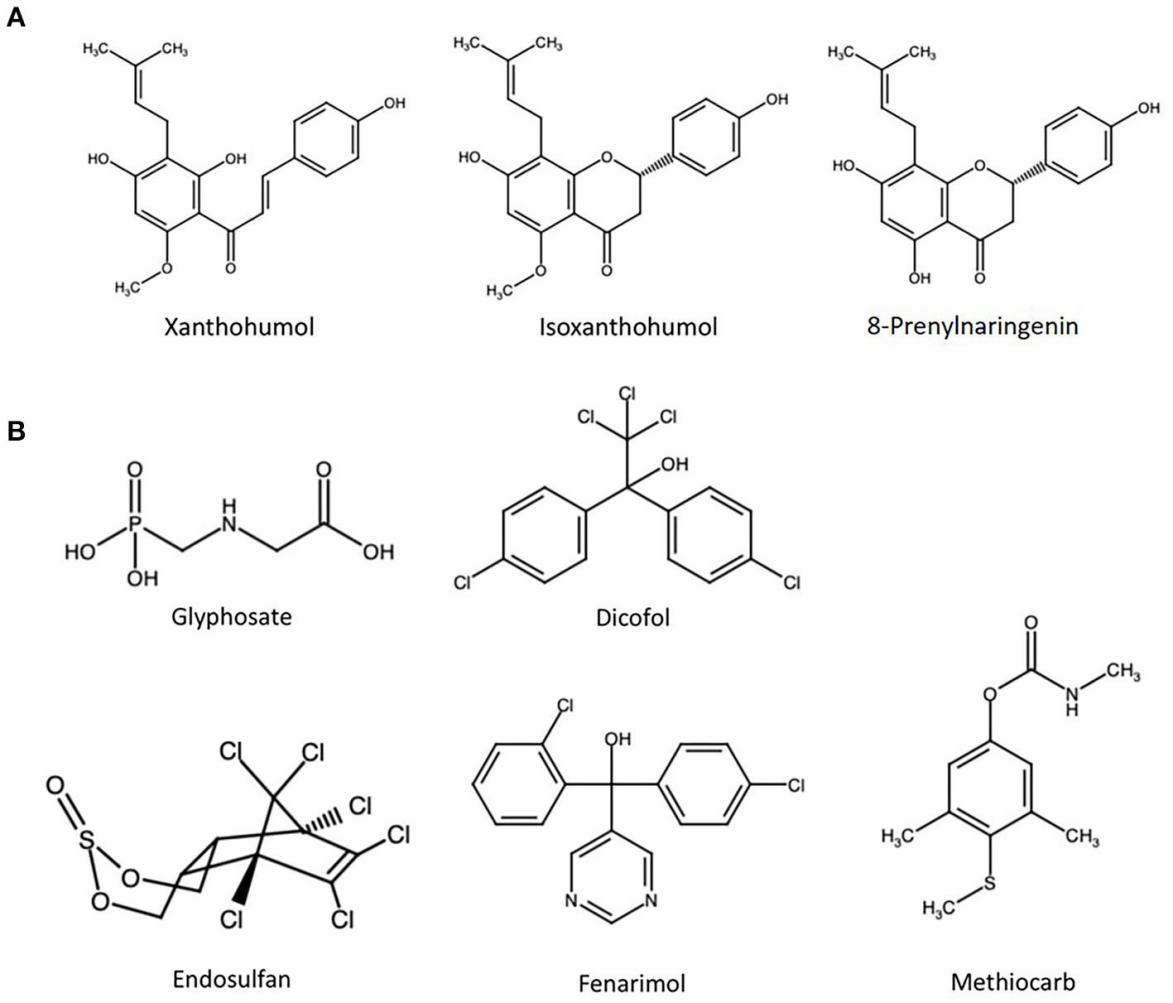
Chemical structure of **(A)** the three most prevalent hop polyphenols in beer and **(B)** selected pesticides with at least one report of estrogenicity.

The latter seem a particularly intriguing compound class from a food toxicologist's point of view. With beer as the main dietary source, prenylated chalcones in general – and the signature compound xanthohumol (XN, Figure 1A) in particular – have been linked with beneficial effects on human health due to their strong anti-oxidative capacity (13). This has also inspired efforts of breweries to develop hop polyphenol-enriched beers (14).

On the other hand, and of potential concern, several metabolites of XN have been described to act as endocrine disruptors (15, 16). Among those compounds, 8-prenylnarginenin (8PN, Figure 1A) stands out as an extremely potent ER agonist and is referred to as the strongest phytoestrogen known today (17). It is formed from the comparably weak xenoestrogen iso-xanthohumol (iX, Figure 1A) *in planta*, but also in the brewing process and during digestion by the gut microbiome (18, 19). Accordingly, hops is widely used in folk and herbal medicine as a treatment for menopausal discomfort (20). It seems somewhat contradictory that prenylated chalcones are on the one hand openly marketed due to their effects on the endocrine system, but on the other hand considered as safe – and even beneficial – in beer. Obviously, this assumption could be valid for toxicokinetic reasons, i.e., when the systemic concentrations of estrogenic hop polyphenols that are achievable from beer consumption are below the point of departure for endocrine disruptive effects. However, this potential issue seems to be not very well-elucidated in a field that has so far largely focused on potentially beneficial properties of flavonoids in beer (13, 21). Moreover, the contribution of hop flavonoids to potential combinatory effects with other xenoestrogens is largely unexplored. In the only study published on this issue, we previously described a mixture of beer polyphenols to antagonize the estrogenic effects of the well-described natural xenoestrogen zearalenone in human endometrial cancer cells, and attributed this effect to anti-estrogenic properties of XN (12).

In the present study, five exemplary pesticides (Figure 1B) were selected based on at least one published report of them acting as ER agonists, among them the publicly discussed herbicide glyphosate (GLP). Alkaline phosphatase (ALP) expression in Ishikawa cells was used as a natural reporter gene system for estrogenicity. On the contrary to some other *in vitro* test systems, this human endometrial cancer cell line expresses both, ER-α and ER-β, and thus facilitates the observation of interactions between chemicals acting as agonists to different receptor isoforms (22). Measuring the ALP-catalyzed conversion rate of 4-nitrophenyl phosphate to 4-nitrophenol as a marker for enzyme expression and thus ER activation, the test compounds were tested individually and in combination to generally address the concern of cumulative effects within this class of pesticides. To enable a direct comparison of effect strength, the same test system was used to determine the estrogenicity of a mixture of the most important hop polyphenols, resembling their respective concentration ratios in beer. Lastly, the two mixtures were combined in different ratios to assess potential combinatory effects toward ER activation.

## Materials and Methods

### Materials

17β-estradiol (E2), 4-nitrophenyl phosphate, diethanolamine, fulvestrant (ICI 182,780), 8-penylnaringenin (8PN), dicofol (Dic), endosulfan (End), fenarimol (Fen), glyphosate (Glp) and methiocarb (Met) were purchased from Sigma-Aldrich (Schnelldorf, Germany), xanthohumol (XN) and iso-xanthohumol (iX) from Extrasynthese (Genay, France). Concerning cell culture, flasks and dishes were obtained from Sarstedt (Nümbrecht, Germany), media (DMEM, DMEM/F-12) as well as fetal bovine serum (FBS) from GIBCO / Thermo Fisher Scientific (Karlsruhe, Germany) and penicillin/streptomycin (P/S) as well as carcoal dextrane-stripped FBS (CD-FBS) from Sigma-Aldrich (Schnelldorf, Germany).

### Cell Culture

The human endometrial adenocarcinoma cell line Ishikawa was obtained from EACC (Salisbury, UK) and cultured in Dulbecco's Modified Eagle Medium (DMEM) supplemented with 5% FBS and 1% P/S, at 37°C, 5% CO_2_ and under humidified conditions. Cells were subcultivated twice a week at a confluence of ~85% and used for experiments at a passage number between 5 and 20 to ensure reproducibility of results.

### Alkaline Phosphatase (ALP) Assay

ALP assays were performed as recently described (23). Briefly, 10,000 Ishikawa cells per well were seeded in 96-well plates and allowed to attach for 48 h in assay medium (phenol red - free DMEM/F-12 supplemented with 1% (*v/v*) CD-FBS and 1% (*v/v*) P/S). Stock solutions were prepared by solving test compounds in DMSO, which were then diluted to the test concentrations in assay medium, resulting in a final DMSO concentration of 0.1–0.2% (*v/v*) (depending on the number of mixed compounds in incubation solutions, but consistently reflected by the same concentration in the respective solvent control). Cells were incubated with incubation solutions containing the test compounds or combinations thereof for another 48 h. Subsequently, cells were washed with PBS and lyzed by shock-freezing. ALP activity was determined by monitoring the conversion of 4-nitrophenyl phosphate to 4-nitrophenol photometrically at 405 nm with a PerkinElmer Victor 3V^TM^ plate reader. The slope of the obtained dose-response curves was taken as a measure for the ER-dependent induction of ALP expression.

### Sulforhodamine B (SRB) Assay

To ensure that effects observed in ALP assays were not caused by cytotoxicity, SRB cell viability assays were carried out in parallel. As with estrogenicity assays, 10,000 cells/well were seeded in 96-well plates, allowed to grow for 48 h and incubated with test solutions for another 48 h. Afterwards, cells were fixed by addition of 50% (v/v) trichloroacetic acid and 1 h incubation at 4°C. After 2 times washing with tab water and 2 times of washing with 1% (*v/v*) acetic acid, plates were dried overnight, incubated with SRB reagent for 45 min, washed twice with water, twice with 1% acetic acid and again dried overnight. Lastly, 10 mM TRIS base buffer was added to dissolve protein-bound SRB and the absorbance of each well was measured at 570 nm with a PerkinElmer Victor 3V^TM^ plate reader.

### Dose Rationale

Representative pesticides (Dic, End, Fen, Glp, Met) were tested in a mixture ratio that was based on the maximum residue levels (MRL) for beer (corresponding to the MRLs in hops or in barley, whichever is set lower) as required by EU law (Table 1). Thus, one unit “MRL eq.” refers to a mixture of pesticides (Mix P), with the dose of each rounded down to meet a round lot nanomolar concentration (i.e., 50 nM Dic, 100 nM End, 50 nM Fen, 500 nM Glp and 200 nM Met). For hop flavonoids, we estimated average concentrations in beer based on a survey (Table 2) by Stevens et al. (24), with one unit “beer eq.” of referring to a flavonoid mixture (Mix F) of 200 nM XN, 2 μM iX and 100 nM 8PN. The estrogenicity of the two mixtures was assessed by dilution of the mixture in parallel to the contained single compounds.

**Table 1:** MRLs for selected pesticides in hops and barley.

**Product**	**Dicofol**	**Endosulfan**	**Fenarimol**	**Glyphosate**	**Methiocarb**
Barley	0.02^*^	0.05^*^	0.02^*^	20	0.1^*^
Hops	50	0.1^*^	5	0.1^*^	0.1^*^
Legislation	Reg. (EU) 899/2012	Reg. (EU) No 310/2011	Reg. (EU) No 318/2014	Reg. (EU) No 293/2013	Reg. (EC) No 839/2008

* *Set at analytical limit of determination*.

**Table 2:** Prenylflavonoid and -chalconoid contents in hops and beer measured by LC–MS/MS, adapted from Stevens et al. (24).

**Beer**	**Xanthohumol** **[μg/L]**	**Isoxanthohumol** **[μg/L]**	**8-Prenylnaringenin** **[μg/L]**
No. 1: Lager/pilsner	34	500	13
No. 2: Lager/pilsner	9	680	14
No. 3: Lager/pilsner	14	400	17
No. 5: American porter	690	1,330	240
No. 6: American hefeweizen	5	300	8
No. 7: Strong ale	240	3,440	110
No. 8: India pale ale	160	800	39
No. 9: European stout	340	2,100	69
No. 10: European lager	2	40	1
No. 11: European pilsner	28	570	21
No. 12: European pilsner	12	1,060	8
No. 13: Non-alcohol beer	3	110	3
**Mean**			
*in μg/L*	*128.1*	*944.2*	*45.3*
in μmol/L^*^	**0.36**	**2.66**	**0.13**

* *Used for the estimation of average polyphenol concentrations in beer*.

While originally planning to combine the two mixtures at a 1:1 ratio to simulate a worst-case scenario of pesticide contamination in beer, we quickly discovered that the hop polyphenol mixture at 1 beer eq. was too potent in activating our test system to be included in combinatory experiments. Thus, we followed two separate approaches to screen for combinatory effects: In an activity-based approach, the pesticide mixture and the polyphenol mixture were combined in a ratio of 1:500, ensuring an induction of ER-mediated gene expression by both mixtures in the tested concentration range. In a wider screening approach, we combined the mixtures in an array of varying concentrations.

### Combination Index (CI) and Statistical Analysis

To assess potential interactions between compounds in the measured combinations, the Combination Index (CI) model of Chou and Talalay (25) was applied with the CISNE curve fitting adjustments as suggested by García-Fuente et al. (26). In brief, this method uses the mass-action law-derived median-effect equation (MDEE) to linearize dose-response curves of single compounds and combinations thereof. Subsequently, it facilitates the calculation of a parameter (the CI) which is indicative for the type and strength of a combinatory effect. In line with the suggestions by Chou (5), CI < 0.9 was considered to indicate a synergistic interaction, CI > 1.1 an antagonism and 0.9 < CI < 1.1 a nearly additive combinatory effect. To apply the model to ALP assay data, the latter were normalized to both, the solvent control and the positive control (1 nM E2, 100% effect). MDEE fitting and CI calculations were carried out using the CISNE tool (26) and MS Excel. Corresponding graphs and all further statistical calculations (normal distribution, one-way ANOVA, etc.) were carried out in Origin^®^ 2020.

## Results

### Cell Viability

To ensure non-cytotoxicity and thus to exclude false reporting anti-estrogenic effects, SRB cell viability assays were carried out in parallel (Ishikawa cells, 48 h incubation) to ALP assays. The single pesticides Dic, End, Fen and Met caused cytotoxic effects at high concentrations, with the lowest observed effect doses reported in Table 3. iX lead to a loss of cellular protein at the highest applied dose of 100 μM, a concentration which was however not applied in ALP assays. Of note, no cytotoxicity was observed for the highest applied concentrations of the herbicide Glp (up to 500 μM), the polyphenol 8PN (500 nM), or the pesticide and polyphenol mixture (and the combination thereof). The non-cytotoxicity of XN in this experimental setup was previously demonstrated (12).

**Table 3:** Onset of cytotoxicity (lowest observed effect level) of toxic single compounds, as observed in at least three independent SRB assays.

**Compound**	**Dic**	**End**	**Fen**	**Met**	**iX**
LOEL (μM)	50	50	100	100	100

### Estrogenicity of Single Compounds

**Figure 2:**
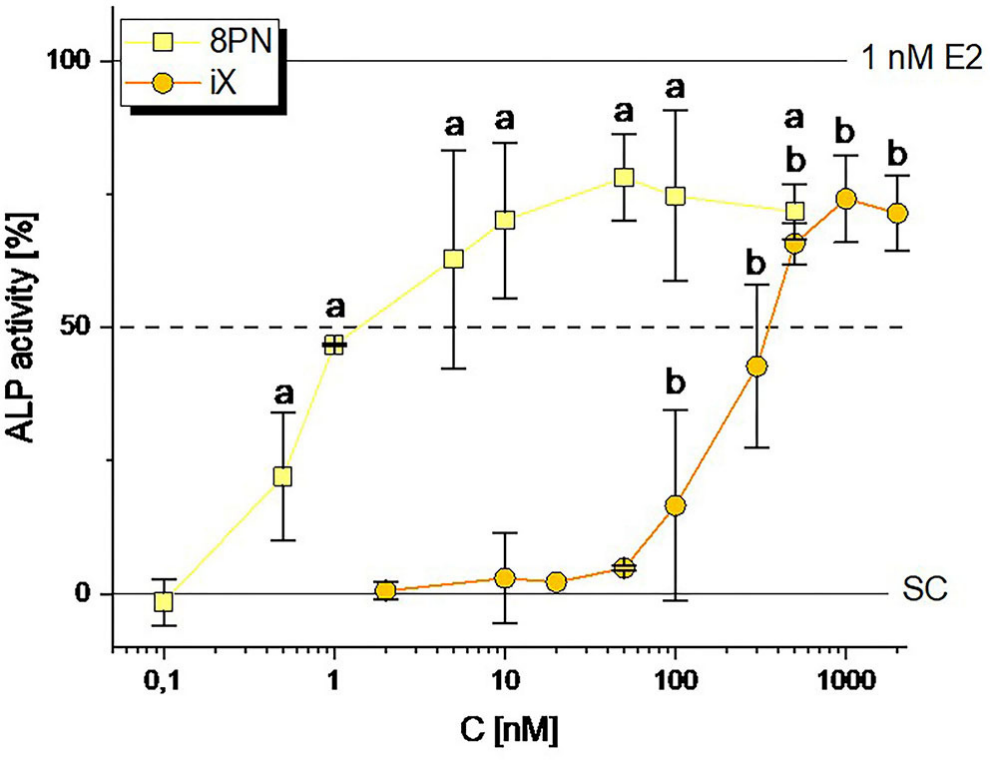
Induction of ALP expression by the hop polyphenols 8-prenylnaringenin (8PN) and isoxanthohumol (iX). Values were scaled to the solvent control (0; 0.1 % DMSO) and the positive control (1 nM E2, 100%) and are expressed as means ± SD of at least 4 independent experiments. Significant differences to the no-effect-level were calculated by one-way ANOVA, followed by Fisher LSD *post-hoc* testing, and are indicated by “a” or “b” (*p* < 0.05). SC, solvent control.

In line with previously published data (12), 8PN was found to induce ALP expression starting from as low as 0.5 nM, with an ED_50_ of 2.44 ± 1.60 nM (Figure 2). The structurally related iX was found to act as a considerably weaker estrogen agonist, with significant ALP induction starting from 100 nM and an ED_50_ of 342 ± 125 nM (Figure 2). Both reached a plateau at about 75% enzymatic activity, thus being confirmed not to fully agonize ER-mediated signaling, in contrast to 1 nM of the natural estrogen E2. XN was previously confirmed not to act estrogenic in this test system (12).

**Figure 3:**
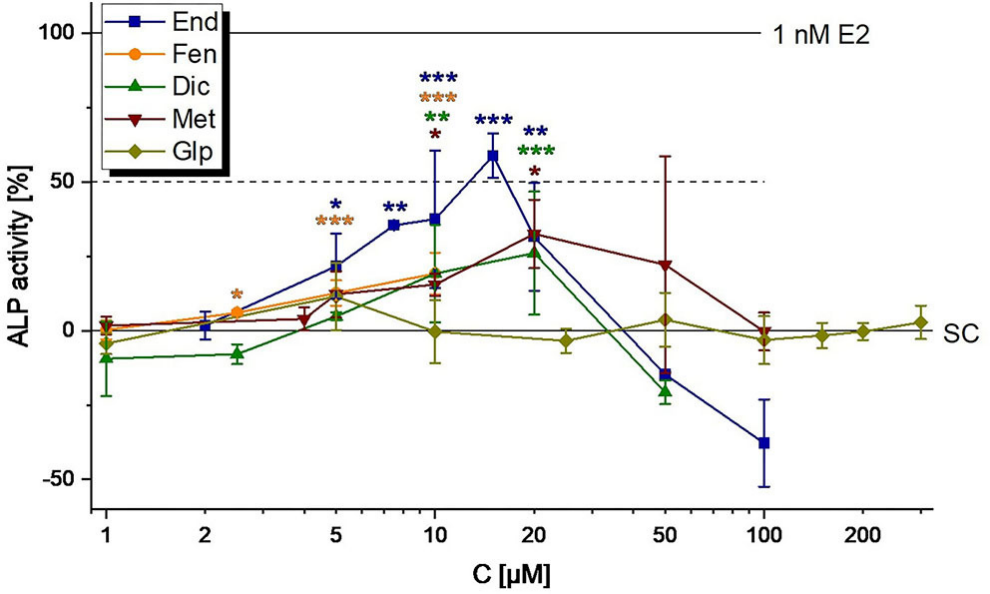
Estrogenicity of representative pesticides (endosulfan, End; fenarimol, FEN; dicofol, Dic; Methiocarb, Met; glyphosate, Glp), as measured in ALP assays. Values were scaled to the solvent control (0%) and the positive control (1 nM E2, 100%) and are expressed as means ± SD of at least three independent experiments. Significant differences to the solvent control (SC) were calculated by an one-way ANOVA test for each compound, followed by Fisher LSD *post-hoc* testing, and are indicated by “*” (p <0.05), “**” (p <0.01) or “***” (*p* < 0.001).

The pesticides End, Fen, Dic and Met were found to induce ALP expression in Ishikawa cells starting from 5, 2.5, 10, and 10 nM, respectively (Figure 3). For all of those, cytotoxic effects quenched estrogenicity at high concentrations (Table 3). Thus, only End was observed to reach the ED_50_ (12.3 ± 2.9 μM, calculated with the CISNE tool). Glp caused neither ER-mediated gene expression nor cytotoxicity in concentrations up to 300 μM (Figure 3). The mediation of all observed effects via ER activation was confirmed by co-incubating active concentrations of the compounds with the selective ER antagonist ICI 182,780 and monitoring the suppression of estrogenicity (Supplementary Figure 1).

### Estrogenicity of Independent Mixtures

**Figure 4:**
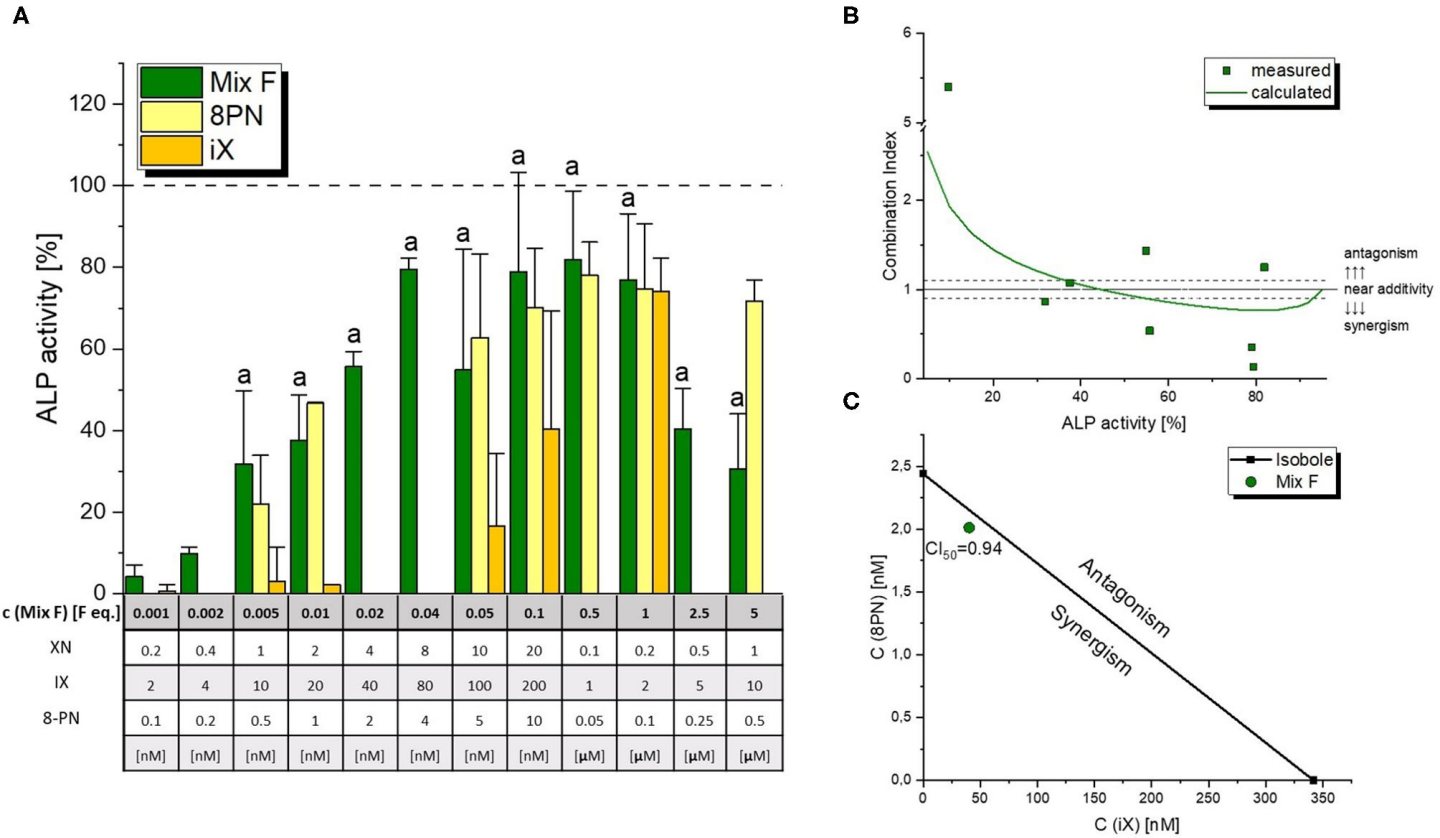
Estrogen receptor dependent induction of ALP expression by a polyphenol mixture. Graph **(A)** depicts ALP assay results for the flavonoid mixture in comparison to contained concentrations of the active constituents 8-prenylnaringenin (8PN) and isoxanthohumol (iX). Values were scaled to the solvent control (0%) and the positive control (1 nM E2, 100%) and are expressed as means ± SD of at least four independent experiments. Significant differences to the solvent control were calculated by one-way ANOVA, followed by Fisher LSD *post-hoc* testing, and are indicated by “a” (*p* < 0.05). The effect-CI plot in graph **(B)** shows the dependency of the combination index on estrogenicity levels, with the line indicating the calculated CI based on the whole dose-response curves and squares representing CI values calculated for actually measured data points. The isobologram in graph **(C)** depicts the strenght of interaction at ED_50_ levels.

The mixture of 8-PN, iX and XN was found to cause ER-mediated ALP expression starting at concentrations as low as 0.005 F. eq. (corresponding to 0.5% of the estimated average dose in beer), and reached a plateau with 0.1 F. eq. (Figure 4A), at about 80% of the activity caused by 1 nM of E2 in line with the testing of single compounds. A strong decline in estrogenicity was observed at concentrations above average beer doses, which could not be linked to cytotoxicity. CISNE/CI analysis revealed a nearly additive cumulative activity of 8-PN and iX at ED_50_ levels, with a CI_50_ of 0.94 (Figure 4B). Of note, the model predicted a slightly synergistic interaction in the higher effect range (~60-80% effect, Figure 4C).

**Figure 5:**
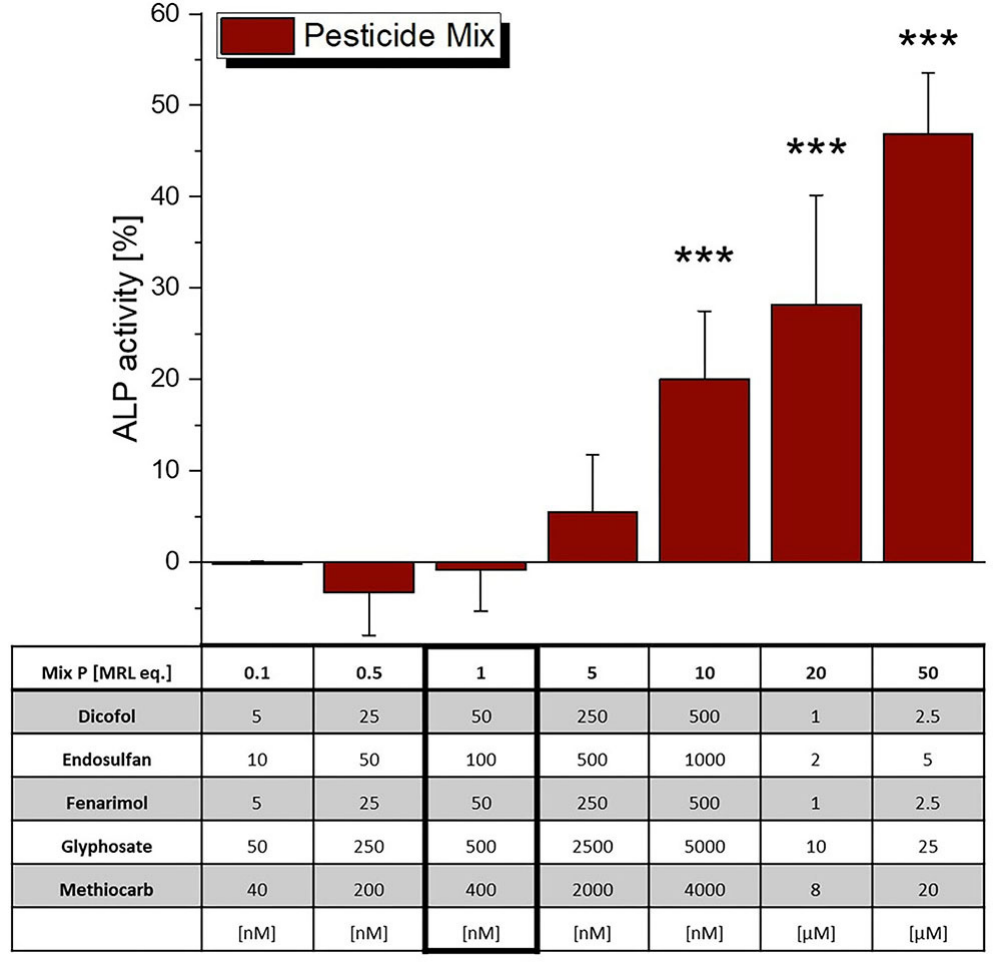
Estrogenicity of a mixture of selected pesticides, as measured in ALP assays. Values were scaled to the solvent control (0%) and the positive control (1 nM E2, 100%) and are expressed as means ± SD of at least 3 independent experiments. Significant differences to the solvent control were calculated by an one-way ANOVA test for each compound, followed by Fisher LSD *post-hoc* testing, and are indicated by “***” (*p* < 0.001).

The incubation of Ishikawa cells with a mixture of the five selected pesticides at their MRL did not lead to significant estrogenic effects in the ALP assay (Figure 5), which were however observed at higher concentrations, starting from 10 MRL eq. Of note, as most of the single compounds did not reach the ED_50_ activity level, CISNE/CI analysis was not applicable for this mixture.

### Combinations of Pesticides and Hop Polyphenols

**Figure 6:**
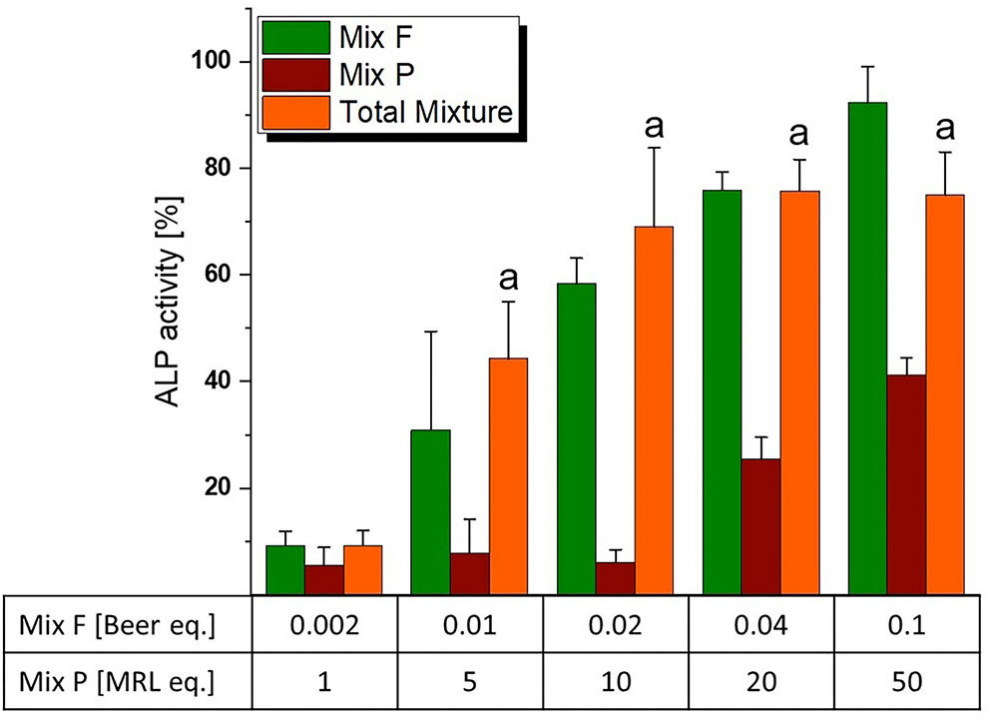
Estrogenicity of the total mixture (comprised of “Mix F” – hop flavonoid mixture and “Mix P” – pesticide mixture), as measured with ALP assays. Values were scaled to the solvent control (0%) and the positive control (1 nM E2, 100%) and are expressed as means ± SD of at least five independent experiments. Significant differences of the total mixture to the solvent control were calculated by one-way ANOVA, followed by Fisher LSD *post-hoc* testing, and are indicated by “a” (*p* < 0.05).

**Figure 7:**
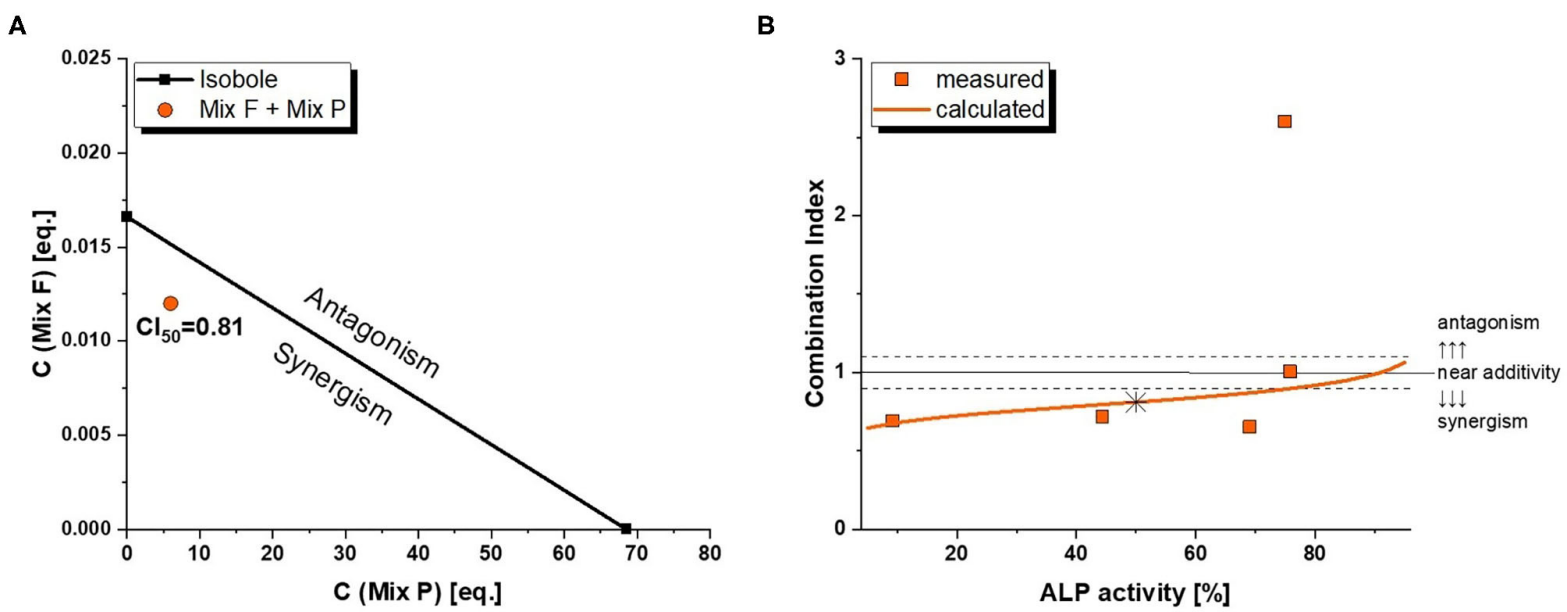
Combination Index (CI) analysis of total mixture estrogenicity. Graph **(A)** depicts the isobologram indicating a slight synergism at ED_50_ levels. The effect-CI plot in graph **(B)** depicts the course of interactive strength in dependence of effect strength.

As mentioned in the dose rationale section, the pesticide and flavonoid mixture were combined in two distinct ways, an activity-based and a wider screening approach. In the former, we applied a constant concentration ratio of 0.002 Beer eq. to 1 MRL eq. Significant induction of ALP expression was observed at concentrations of 0.01/5 eq. and higher (Figure 6). Of note, the combination was slightly – albeit not significantly–more active than the flavonoid mixture even at concentrations where Mix P was still inactive. This was reflected in combination index analysis (Figure 7A), where a CI_50_ of 0.81 was calculated, indicating a slightly synergistic interaction of the two mixture toward ER activation. Moreover, effect-CI plotting (Figure 7B) revealed a synergistic interaction over most of the observable effect range.

**Figure 8:**
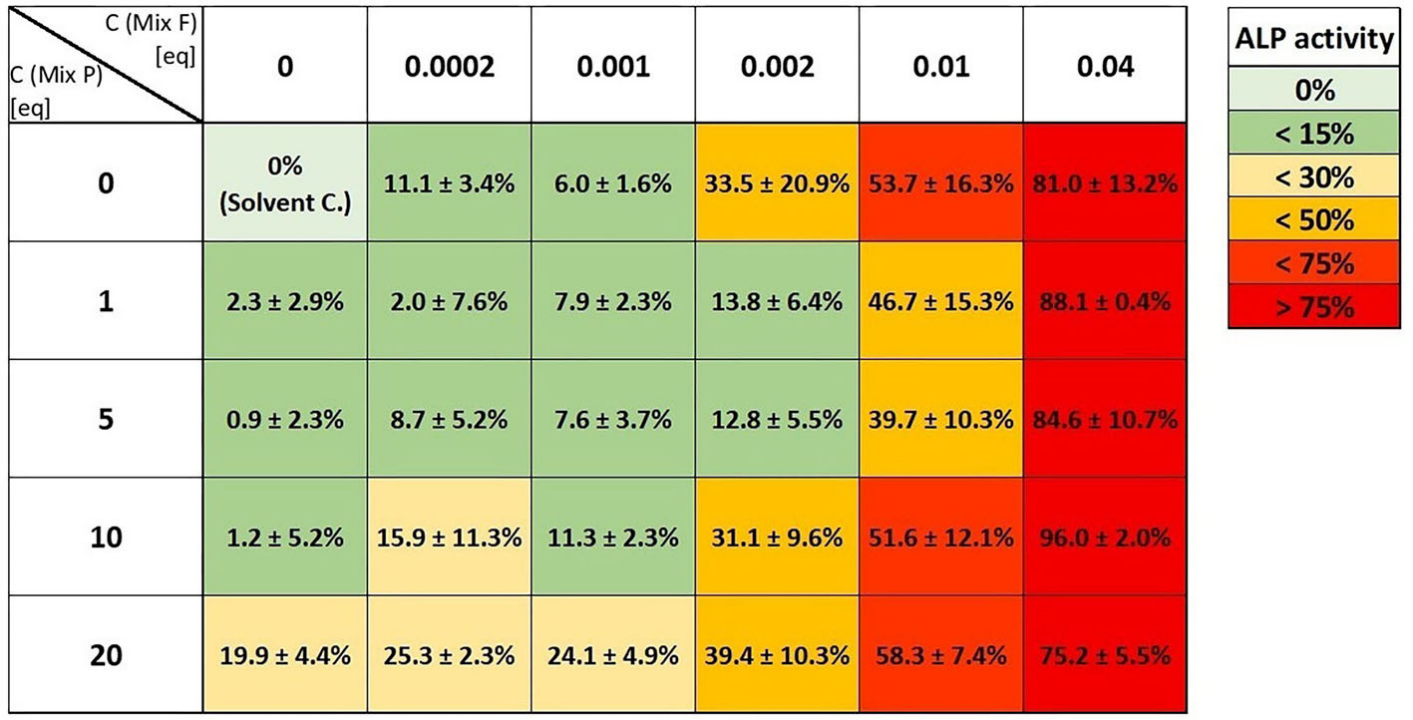
Heat map analysis of the screening approach to assess the combinatory estrogenic effects of combination of the flavonoid mixture (“Mix F”) and the pesticide mixture (“Mix P”) in an array of different concentrations. Values (normalized to both, solvent and positive control) are given as means ± SD of at least three independent experiments.

In the screening approach, we combined the two mixtures in an array of different concentrations to investigate a potential influence of combination ratios more broadly (Figure 8). Here, no synergistic interaction was apparent, apart from a few combinatory incubations that gave higher signals than the corresponding concentration of the single mixture (e.g., the combination of 10 eq. Mix P with 0.0002 or 0.001 eq. of Mix F).

## Discussion

Reports that constituents from hops are able to cause endocrine disruption date back to the middle ages, where German clergy added the plant to beer not only for conservation, but also to lower the libido (27). Later, menstrual disturbances of hops-harvesting women were commonly described to occur, and folk and herbal medicine use hop extracts to treat menopausal disorders. Milligan et al. (15) offered an explanation for these phenomena, as they identified the prenylated chalcone 8PN as an extremely potent phytoestrogen. Keeping all this in mind, it seems odd that the occurrence of 8PN and other estrogenic metabolites of the parent flavonoid XN in beer has not drawn more concern, particularly in a time where the hazard of xenoestrogens from man-made sources like pesticides or bisphenol A are publicly discussed. On the opposite, it seems that the scientific discourse is largely focused on potential beneficial effects of hop polyphenols, particularly their anti-oxidative properties (13) and their potential as anti-diabetic agents (28) while neglecting their potential as endocrine disruptive chemicals. This has even inspired breweries to strive to enrich prenylated chalcones in beers (14).

An often-used justification for discarding endocrine disrupting phytoestrogens in beer as a health concern is that the low bioavailability of hop polyphenols would limit systemically achieved doses to non-hazardous concentrations. At first sight, this notion seems to be supported by pharmacokinetic studies. In a human intervention study administering 20 mg of XN, a peak plasma concentration (C_Max_) of 33 ± 7 μg/L and an area under the curve (AUC) value of 92 ± 68 h×μg/L was reported (29), from which one could roughly estimate a low bioavailability of ~2% by assuming standard physiological parameters. However, the bioavailability of 8PN might be much higher as compared to XN, with van Breemen et al. (30) reporting an AUC of 24.3 ± 8.9 h×μg/L and a C_Max_ of 1.4 ± 0.3 μg/L (= 3.8 nM) in humans receiving an extract from hops containing just 0.25 mg 8PN, which would corresponds to the concentration of 8PN per liter in strongly hopped beers. This might partially result from the additional formation of 8PN from the naturally co-occurring iX, the most prevalent prenylflavonoid in beer (24), within the human gut (18) and liver (31). This reaction should be regarded as a metabolic activation due to the much higher estrogenicity of the reaction product. Assuming linear kinetics, the consumption of 0.5–1 L of average-hopped beer (Table 2) could result in systemic 8PN concentrations around 0.5 nM, a dose sufficient to cause significant estrogenicity in our *in vitro* test system (Figure 2).

Additionally, we hereby report a cumulative to slightly synergistic activity of iX and 8PN toward the activation of ERs (Figure 4). In fact, the mixture of iX, 8PN and XN was able to significantly induce estrogenicity at a concentration of 0.005 beer eq., corresponding to a 200 times lower concentration of those compounds than estimated to occur in an average beer. At 0.04 beer eq., the ALP assay system was fully induced. These findings suggest cumulative/synergistic combinatory effects likely to occur under realistic exposure scenarios. Additional metabolites, e.g., 6-prenylnaringenin, that were not included in our study but were described to act estrogenic (16), should also be expected to cumulatively enhance the effects of 8PN as a leading compound in a similar way than demonstrated by our results. Thus, we suggest that risk assessment should avoid focusing on isolated compounds but should rather account for the simultaneous uptake of several endocrine active prenylated chalcones.

Of note, estrogenicity declined at high concentrations of the hop polyphenol mixture (Figure 4), an effect that could not be attributed to cytotoxicity (Table 3) and further confirms an anti-estrogenic activity that was previously described for XN (12, 32). As an antagonism toward the ER can also be considered a pathway of endocrine disruption, one might consider including XN in respective toxicological studies.

Another issue might arise from the proposed pharmaceutical use of hop flavonoids in the treatment of menopausal complaints (33) that could lead to the circulation of much higher doses of 8PN and related phytoestrogens as compared to nutritional exposure. Potentially adverse effects arising from prenylated flavonoids should be fully elucidated before pursuing these approaches. As reviewed by Bolton et al. (34), several intriguing biological pathways have been described to be targeted by those polyphenols, e.g., the activation of AhR receptors that is also discussed as a mechanism for potential endocrine effects of other natural compounds (35), or the activation of the Nrf2 pathway, a cellular defense system against oxidative stress. However, a complete toxicological characterization of the activities *in vivo* is still lacking.

On the opposite to hop flavonoids, the representative pesticides tested in this study exhibited much less estrogenic activity. At concentrations corresponding to the MRL level, none of the compounds induced the *in vitro* test system (Figure 3). In line with previous studies (36–38), the compounds Dic, End, Fen and Met caused estrogenicity at higher micromolar levels. Of note, Glp did not act estrogenic in Ishikawa cells up to a concentration of 300 μM, a result which is contradictory to the work of Mesnage et al. (39) who described an estrogen-like growth stimulation in MCF-7 breast cancer cells at concentrations ≥ 59 μM. This discrepancy could hint at a tissue-specific response to the herbicide, which should encourage further investigations, particularly on the *in vivo* situation, to clarify a potential endocrine impact of the widely-used herbicide.

The mixture of the above stated pesticides did not cause ER-mediated ALP expression at a level corresponding to the MRL (1 MRL eq.), but only at 10 times elevated concentrations (Figure 5). Thus, potential synergistic interactions that could put effective MRL levels into question were not observed, which seems to confirm their toxicological soundness.

Despite the pesticide mixture not being active at 1 MRL eq., it interacted in a slightly synergistic way with the hop flavonoid mixture (Figure 7). As humans are typically not exposed to isolated xenoestrogens, but to a mixture of vastly different chemicals from different (both natural and man-made) sources, this further underlines the importance of considering mixture effects in toxicological risk assessment. Given that the endocrine activity of hop phytoestrogens occurs at concentrations that could potentially be achieved by common nutritional exposure - the average beer consumption per capita is above 100 L/year in some regions of Europe (40) – synergistic estrogenic interactions with these compounds seem to be of high relevance and should be explored with further co-occurring substances.

## Conclusion

Taken together, this study points at the possible underestimation of the endocrine activity of prenylated chalcones found in beer due to cumulative and synergistic mixture effects, and demonstrates the high potential for naturally occurring EDCs to synergistically interact with co-occurring compounds from artificial sources, a topic which should be increasingly considered in risk assessment.

## Data Availability Statement

The raw data supporting the conclusions of this article will be made available by the authors, without undue reservation.

## Author Contributions

GA was involved in study design, statistical analysis, supervised experiments, and wrote the manuscript. GB carried out experimental work and refined the manuscript. DM was involved in study design, supervised the project, and refined the manuscript. All authors contributed to the article and approved the submitted version.

## Conflict of Interest

The authors declare that the research was conducted in the absence of any commercial or financial relationships that could be construed as a potential conflict of interest.
