# Supplementary material for: Systemically Achievable Doses of Beer Flavonoids Induce Estrogenicity in Human Endometrial Cells and Cause Synergistic Effects With Selected Pesticides
Source: Front Nutr. 2021 Jun 7;8:691872. doi: 10.3389/fnut.2021.691872 (PMC8215115; doi:10.3389/fnut.2021.691872)
Supplement: Supplementary file 1 [file Table_1.DOCX]

Supplementary figures

Figure S1: ALP activity after incubation with single pesticides (dark grey) and the suppression thereof by co- incubation with the ER antagonist ICI 182,780. Values are expressed in relation to the solvent control (0.1% DMSO) and as means + SD of at least three independent experiments. Significant differences to the respective incubation with the single compound were calculated by Student’s *t*-test and are indicated by “*” (p<0.05), “**” (p<0.01) or “***” (p<0.001)
