# Supplementary figures and images for: Systemically Achievable Doses of Beer Flavonoids Induce Estrogenicity in Human Endometrial Cells and Cause Synergistic Effects With Selected Pesticides
Source: Front Nutr. 2021 Jun 7;8:691872. doi: 10.3389/fnut.2021.691872 (PMC8215115; doi:10.3389/fnut.2021.691872)

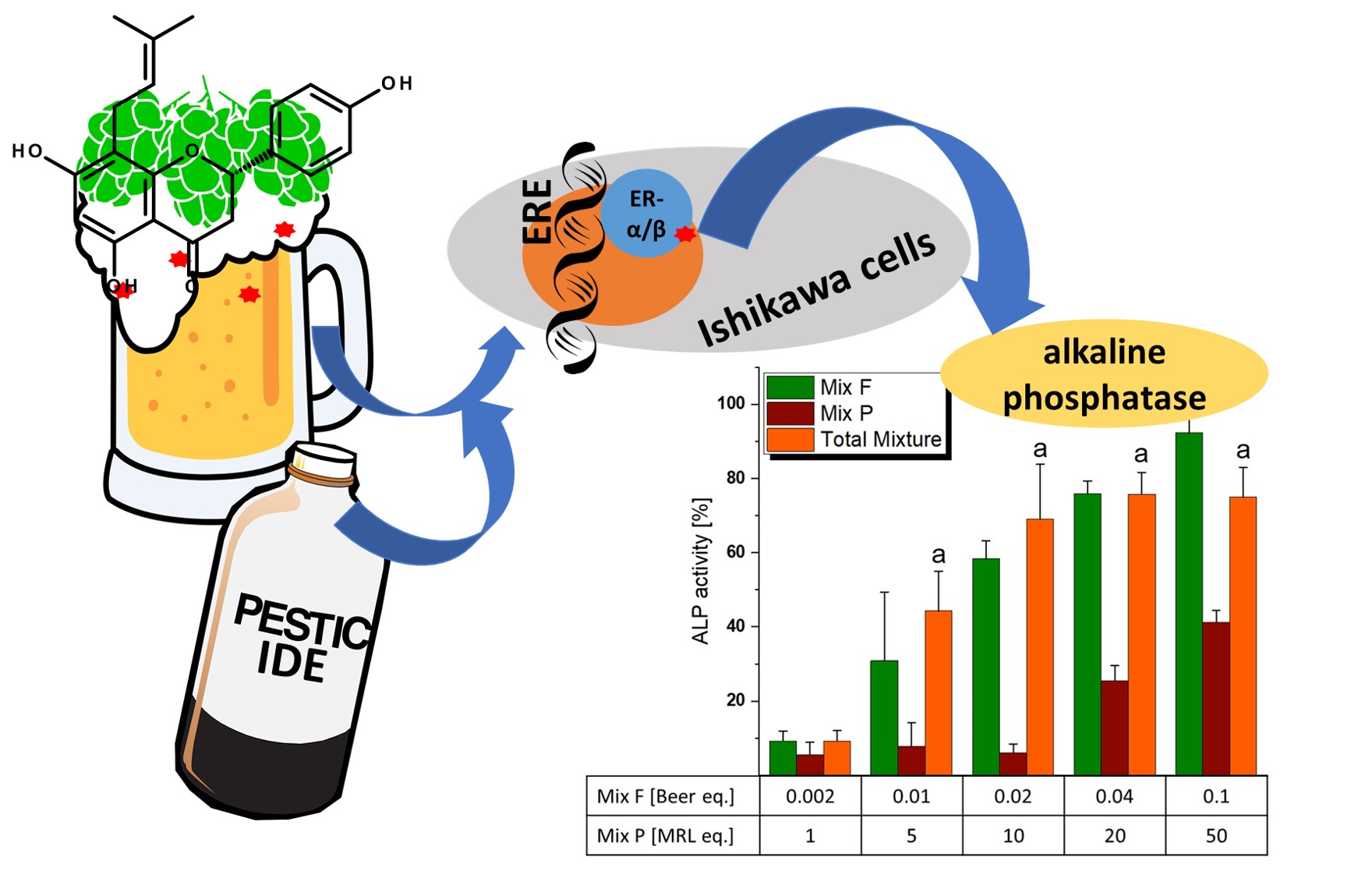

Supplement: Supplementary file 2 [file Image_1.JPEG]
